# Supplementary figures and images for: The pluripotent stem cell-specific transcript ESRG is dispensable for human pluripotency
Source: PLoS Genet. 2021 May 25;17(5):e1009587. doi: 10.1371/journal.pgen.1009587 (PMC8184003; doi:10.1371/journal.pgen.1009587)

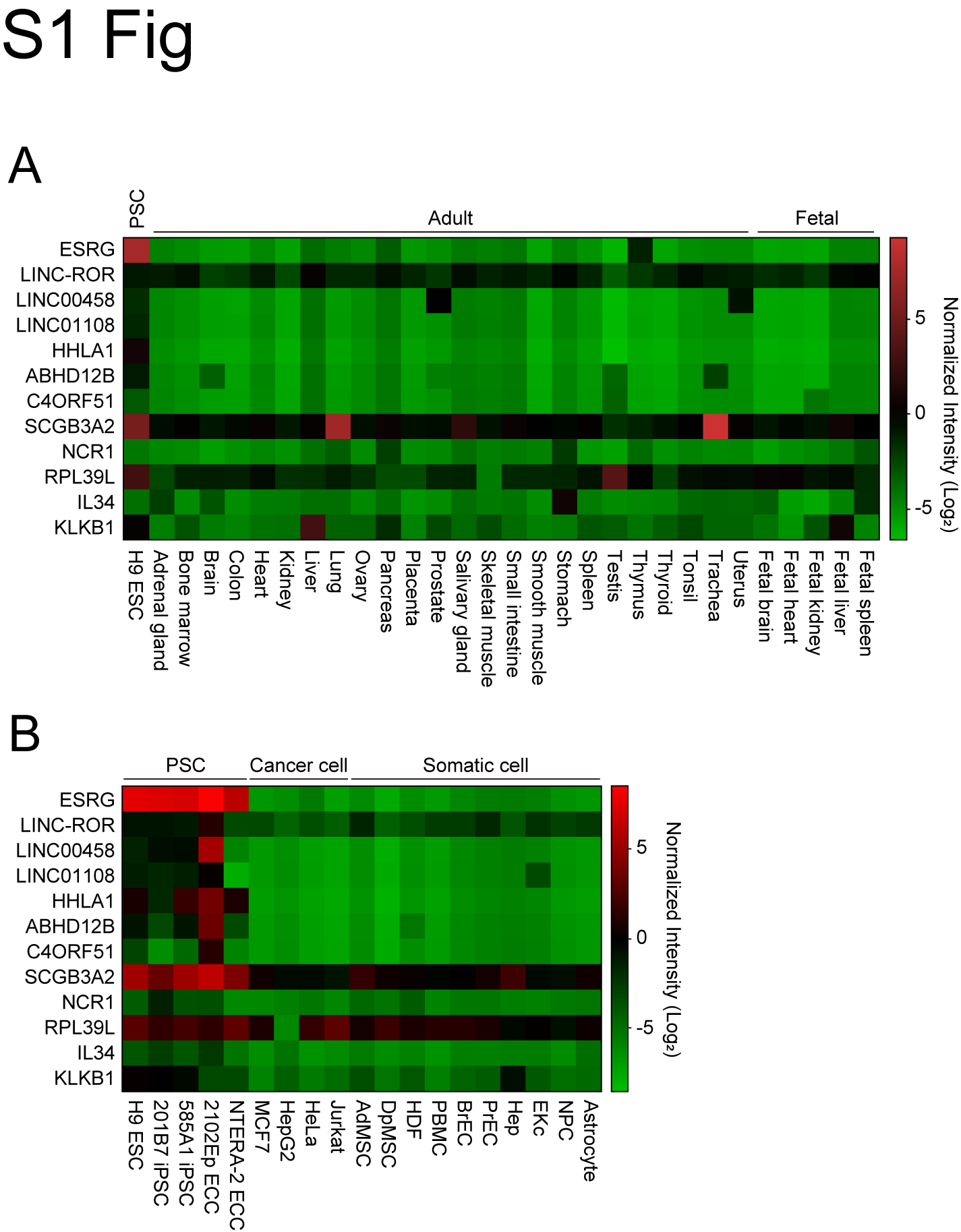

Supplement: S1 Fig — Expression of ESRG in human tissues. (A) Shown are the normalized intensities of ESRG expression from the microarray data of PSC (H9 ESC), 24 human adult tissues, and five fetal tissues. (B) Expression of ESRG in human cell lines. The normalized intensities of ESRG expression from the microarray data of several PSC lines including H9 ESC, 201B7 iPSC, 585A1 iPSC, 2102Ep embryonic carcinoma cells (ECC) and NTERA-2 ECC, cancer cell lines such as MCF7, HepG2, HeLa and Jurkat, and normal tissue-derived cells such as adipose tissue-derived mesenchymal stem cells (AdMSC), dental pulp-derived MSCs (DpMSC), human dermal fibroblasts (HDF), peripheral blood mononuclear cells (PBMC), bronchial epithelial cells (BrEC), prostate epithelial cells (PrEC), hepatocytes (Hep), epidermal keratinocytes (EKc), neural progenitor cells (NPC) and astrocytes (Astrocyte) are shown. Numerical values for A and B are available in S1 Data. (TIF) [file pgen.1009587.s001.tif]

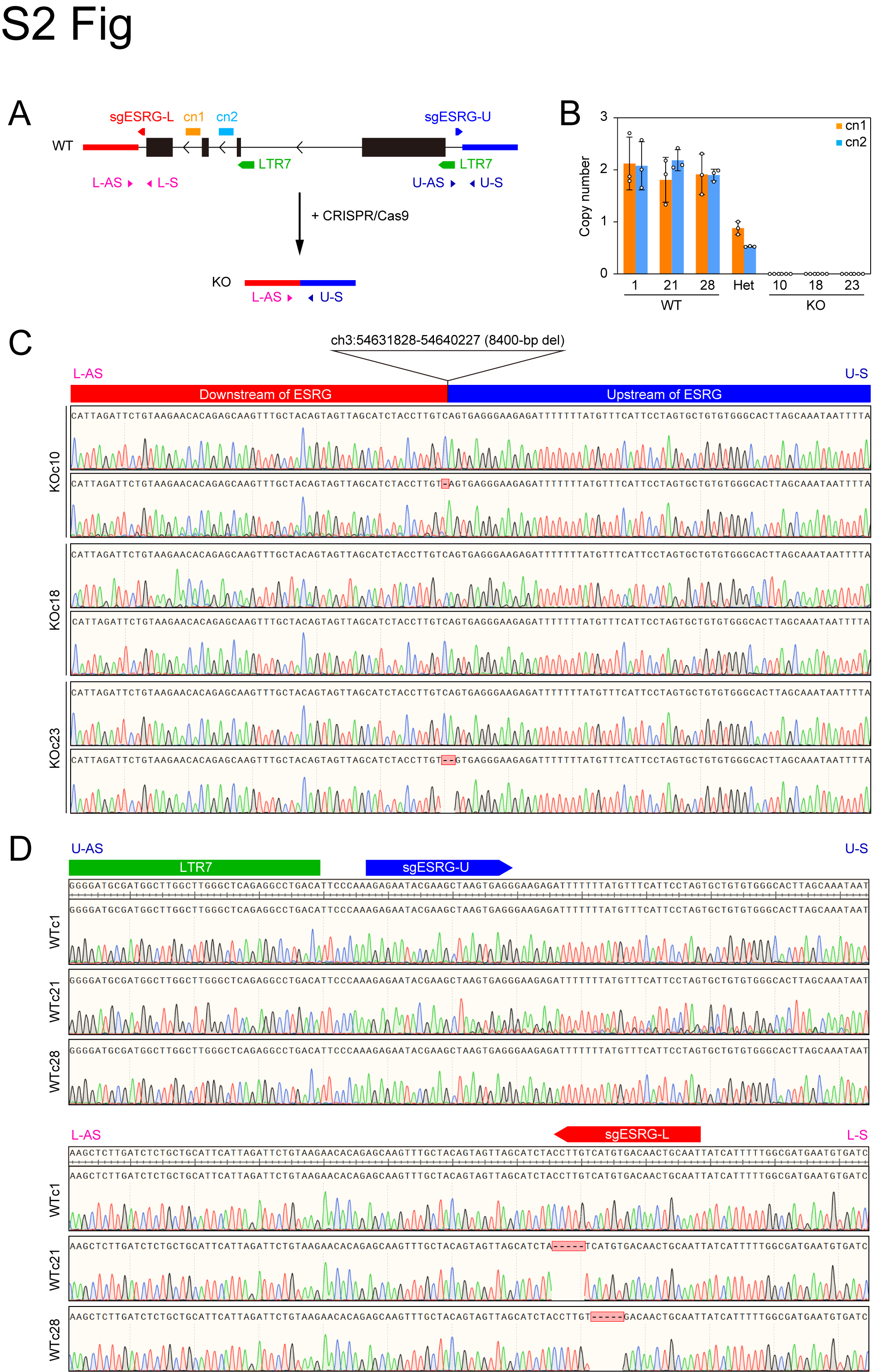

Supplement: S2 Fig — (A) The scheme of ESRG targeting. The locations of sgRNAs for targeting (sgESRG-U and -L), primers for genotyping (U-S/AS and L-S/AS) and TaqMan Assays for copy number analyses (cn1 and cn2) are shown. The sequences of sgRNAs and primers are provided in the Methods section and S5 Table. (B) The copy number of the ESRG gene. The copy number of ESRG gene in ESRG WT (clones 1, 21 and, 28), a heterozygous clone (Het) that lacks one ESRG allele and KO (clones 10, 18 and, 23) were quantified by qPCR using TaqMan Copy Number Assays (cn1 and 2). Values are normalized by RNase P and compared with parental H9 ESCs. n = 3. (C) The sequences around the deletion sites in ESRG KO ESC clones verified by Sanger sequencing. (D) The sequences around the sgRNA recognition sites upstream (sgESRG-U) and downstream (sgESRG-L) of the ESRG locus in ESRG WT ESC clones verified by Sanger sequencing. Numerical values for B are available in S1 Data. (TIF) [file pgen.1009587.s002.tif]

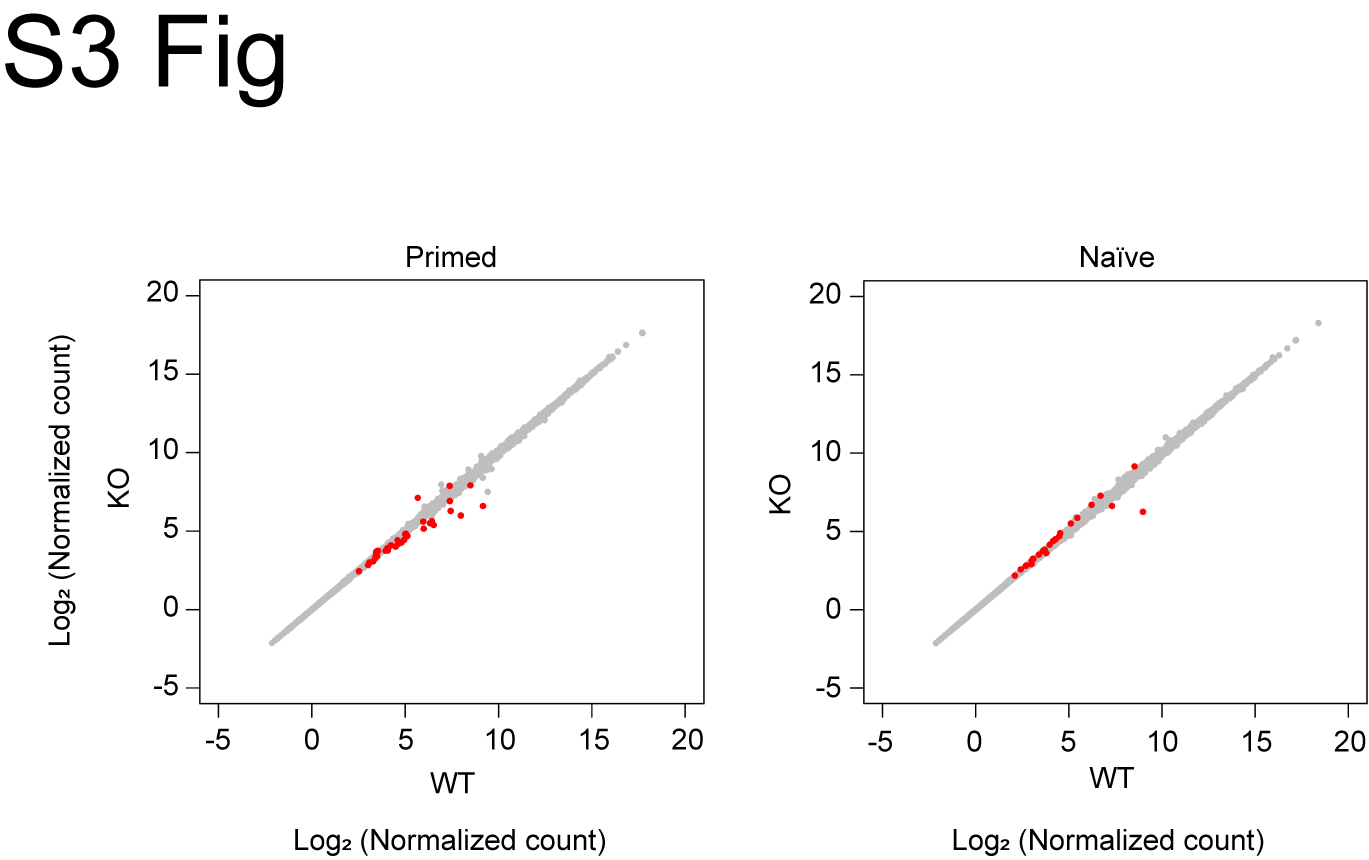

Supplement: S3 Fig — Global gene expression. Scatter plots compare log2 (Normalized count) of the RNA-seq data of ESRG WT and KO primed (left and naïve (right) PSCs. The colored plots indicate differentially expressed genes (DEGs) with statistical significance (FC>2.0, adjusted p-value <0.05). Three clones of ESRG WT and KO PSCs at different three passage numbers were analyzed in each condition. (TIF) [file pgen.1009587.s003.tif]

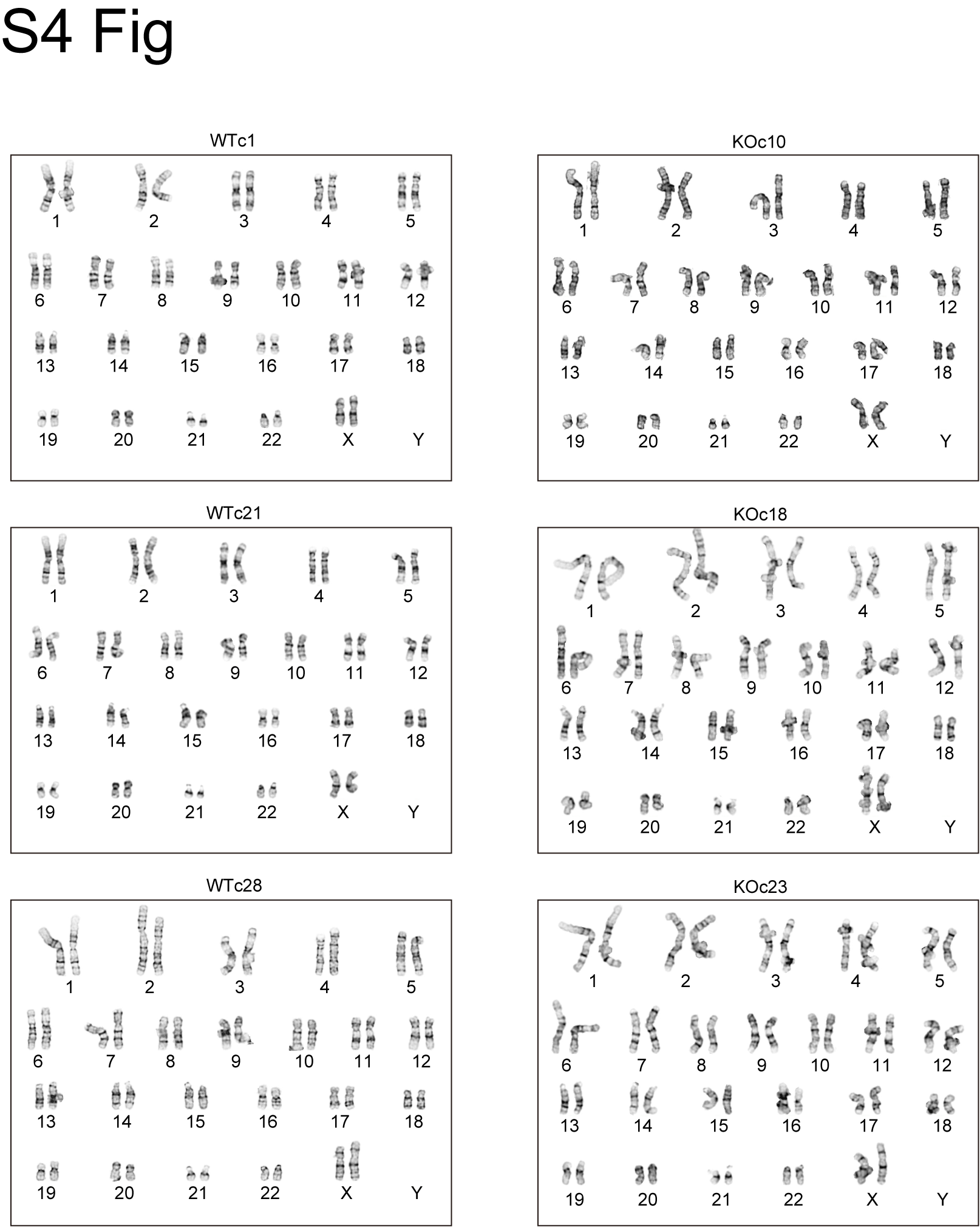

Supplement: S4 Fig — Representative images of G-band staining show that all clones used in the study maintained normal female karyotypes (46XX). (TIF) [file pgen.1009587.s004.tif]

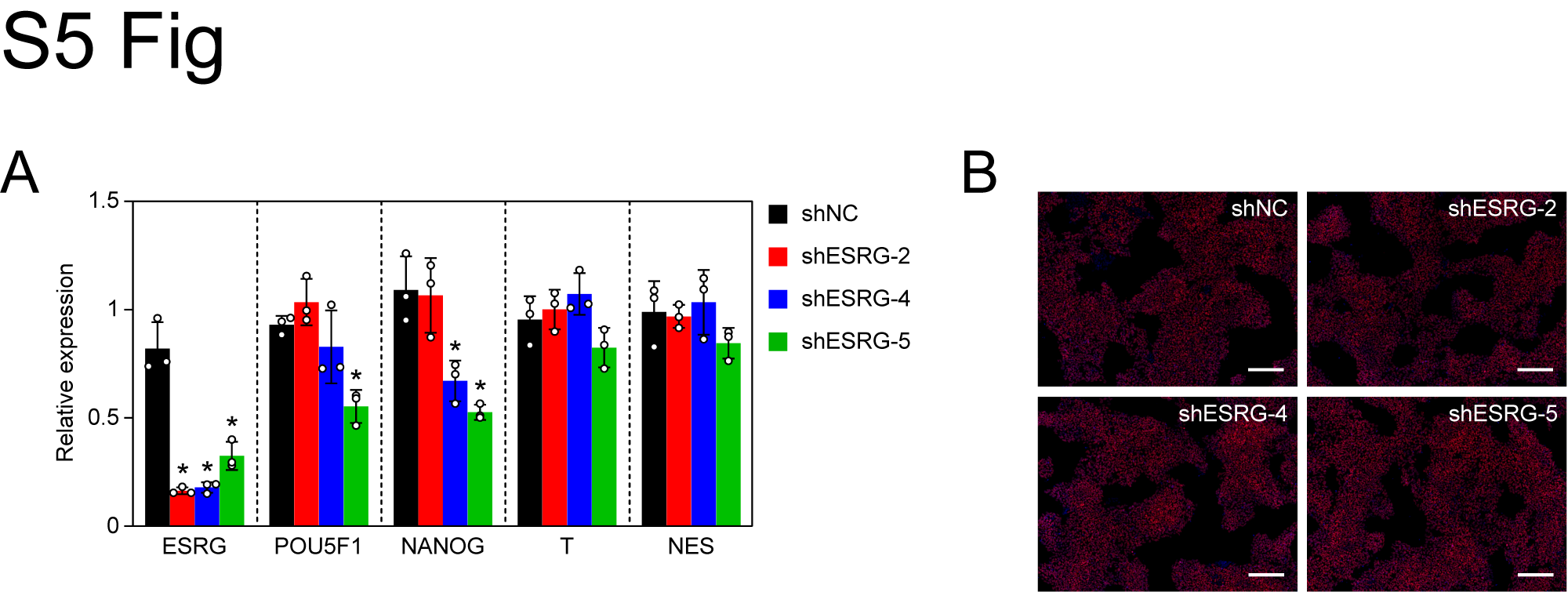

Supplement: S5 Fig — (A) Shown are relative expressions of ESRG, POU5F1, NANOG, T, and NES in primed H9 ESCs transduced with empty vector (shNC), and shRNAs against ESRG (2, 4, and 5). Values are normalized by GAPDH or ACTB and compared with the primed H9 ESC line. *P<0.05 vs. primed H9 ESC line by unpaired t-test. n = 3. (B) Representative images of ESRG KD cells of immunocytochemistry for NANOG. Bars, 200 μm. Numerical values for A are available in S1 Data. (TIF) [file pgen.1009587.s005.tif]

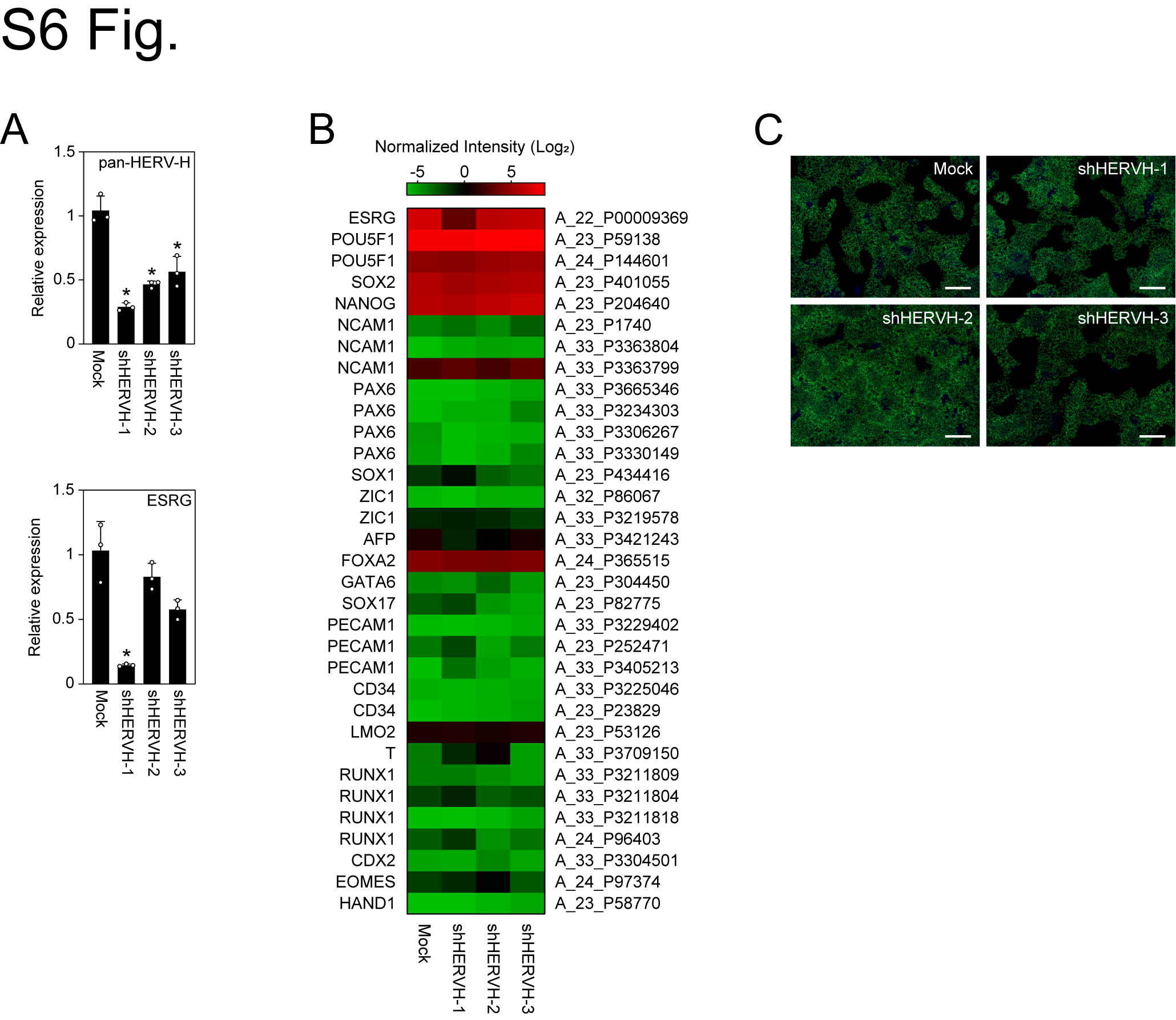

Supplement: S6 Fig — (A) The KD efficiencies of pan HERV-Hs. Shown are relative expressions of pan HERV-Hs and ESRG in primed N18 iPSCs transduced with empty vector (Mock), and shRNAs against HERV-Hs (1, 2 and 3). Values are normalized by GAPDH and compared with the primed N18 iPSC line. *P<0.05 vs. primed N18 iPSC line by unpaired t-test. n = 3. (B) The expression of PSC and differentiation markers in HERV-H KD cells. The heatmap shows the normalized intensity of the indicated genes analyzed by microarray. Each value is the average of biological triplicates. (C) Representative images of HERV-H KD cells of immunocytochemistry for NANOG. Bars, 200 μm. Numerical values for A and B are available in S1 Data. (TIF) [file pgen.1009587.s006.tif]
