# Supplementary material for: The pluripotent stem cell-specific transcript ESRG is dispensable for human pluripotency
Source: PLoS Genet. 2021 May 25;17(5):e1009587. doi: 10.1371/journal.pgen.1009587 (PMC8184003; doi:10.1371/journal.pgen.1009587)
Supplement: Attachment — Submitted filename: Answer to reviewers comments_04262021.docx [file pgen.1009587.s014.docx]

Dear reviewers,

We sincerely appreciate the valuable comments. We answer all comments given by reviewers #1 and 2 in a point-by-point manner. The black and red letters indicate the reviewers’ comments and our answers, respectively.

**Reviewer #1:** In this manuscript, the authors demonstrated that the pluripotent stem cell-specific lncRNA ESRG is dispensable for human PSC self-renewal and lineage commitment, and reprogramming of neural stem cells to iPSCs.

It has been previously shown that ESRG knockdown causes hESC differentiation and overexpression enhanced reprogramming efficiency (Wang, et al., Nature, 2014). The authors demonstrated data that contradict with this previous report in Nature by using total deletion of ESRG gene with two gRNAs together with Cas9.

The data are solid and it is unfortunate that the authors needed to spend time to demonstrate it was ‘not’ important. It might be interesting to test exactly the same shRNA used by Wang, et al. to see its effect on PSC self-renewal and overexpression of ESRG in fibroblast reprogramming as Wang, et al. did. It is also interesting to see the pan-HERV-Hs shRNAs cause hPSC differentiation as another paper reported (Lu, Nat Struct Mol Biol, 2014), in comparison with shLTR7-1 which the authors previously used to revert the differentiation defective iPSC phenotype (Ohnuki, PNAS, 2014). These experiments could confirm whether the previously reported importance of HERV-Hs for hPSCs identity is true or not in these authors’ hands/culture condition. If the bulk HERV-Hs expression is indeed important, ESRG KO hESCs could be a useful tool to investigate how HERV-Hs control gene expression as ESRG KO hESCs have only 10 down-regulated genes? At least some of them could be directly regulated? Are any of the 10 genes closely located to ESRG and cis interaction can be observed?

**The function of ESRG** | In the revised manuscript, we have added the description of 7 differentially expressed genes (10 probes in microarray) such as ESRG (Chr.3), TMLHE (Chr. X), LDHC (Chr. 11), LOC339975 (Chr. 4), AIFM2 (Chr. 10), XLOC_L2_01411 (Chr. 4) and lnc-CDKAL1-1 (Chr. 6) between ESRG WT and KO PSCs in primed condition. We also have added RNA-seq data to validate the results of microarray analyses according to reviewer #2’s suggestion (Figure S3). The RNA-seq data also showed no evidence of cis-regulation of neighbor genes by ESRG.

**Testing ESRG shRNAs** | In the revised manuscript, we have added the results of shRNA-mediated ESRG knockdown as Figure S5. We tested three shRNAs, including a newly designed one in this study (shESRG-2) and two published sequences (shESRG-4 and 5 correspond shESRG #4 and #5 of the previous study by Wang et al., respectively) (PMID: 25317556). These three shRNAs suppressed the ESRG expression at similar levels to the results by Wang et al. (PMID: 25317556). All cell lines were able to expand normally at least 20 days after transduction of shRNAs. As shown in Figure S5A, shESRG-4 decreased NANOG expression, and shESRG-5 decreased POU5F1 and NANOG with statistical significance. In contrast to these two published shRNAs (4 and 5), shESRG-2 changed neither POU5F1 nor NANOG expression. Immunocytochemistry for NANOG shown in Figure S5B showed that all ESRG knockdown cells expressed NANOG protein. Therefore, combined with the knockout data, ESRG is not essential for the self-renewal of human PSCs.

**Overexpression of ESRG in fibroblast reprogramming** | In our previous work published in 2018 (PMID: 29641997), we performed the overexpression of ESRG along with reprogramming factors in human fibroblasts. As a result, ESRG did not promote the reprogramming of human fibroblasts to iPSCs. This study revisited the effects of ESRG on iPSC reprogramming using ESRG WT and KO neural progenitor cells (NPCs). Although we tested if ESRG overexpression enhances reprogramming efficiency, we observed no significant effect of ESRG on reprogramming efficiency. In addition, Figure 5B of the revised manuscript demonstrated that ESRG KO NPCs generated iPSCs. Taken together, therefore, we conclude that ESRG is dispensable for iPSC generation.

**Comparison of pan-HERV-H shRNAs** | We would like to notify that our previous studies (PMID: 25097266; PMID: 32492424) revealed that pan-HERV-H knockdown did not induce spontaneous differentiation of human PSCs like others such as Liu et al. and Wang et al. In the revised manuscript, we have added the results of pan-HERV-H knockdown by using three different sequences (shHERVH-1 by Ohnuki et al., 2014 (PMID: 25097266); shHERVH-2 by Liu et al., 2014 (PMID: 24681886); and shHERVH-3 by Wang et al., 2014(PMID: 25317556)) in human iPSCs (Figure S6). All shRNAs decreased the expression of pan-HERV-Hs (see Figure S6A). The microarray data shown in Figure S6B and immunocytochemistry for NANOG protein suggest that knockdown of HERV-Hs did not induce the differentiation of primed human PSCs.

A recent paper by Zhang et al. (PMID: 31427791) showed that pan-HERV-H knockdown in human PSCs by using CRISPR interference did not induce spontaneous differentiation as we observed. We have added the description regarding the paper in the discussion section.

**Reviewer #2:** In this paper Takahashi and colleagues investigate the effect of deleting the long non-coding RNA ESRG in human pluripotent stem cells. This is interesting as ESRG is driven by an HERV-H element and a good candidate to be one of the functional elements responsible for the previously observed effect on pluripotency of a general HERV-H knock-down. They find that the deletion does not result in any major pluripotency phenotype. Although this is a negative finding, I still find this result interesting and relevant as it is 1) generally important to publish negative results, 2) the analysis is overall technically sound and carefully conducted and 3) it is an “unexpected” (line 43) result from a classically biochemical/mechanistical viewpoint, but I do not find it unexpected from an evolutionary viewpoint.

However, the paper would greatly profit from some improvements:

Major concerns:

1. The most important in my view, is the analysis of the gene expression data from the 3 WT and 3 KO lines. I think 3 replicates are just not enough to infer changes in gene expression robustly. Furthermore there are apparent expression changes (Fig. 1G), but they are not discussed at all. I think a proper analysis would involve RNA-seq data and not microarray data as the false negative rate can be much better estimated for RNA-seq. Additionally, it would take ideally more clones as biological replicates or – if that is not readily available - at least independently grown replicates of the clones (e.g. 3 per clone), to have at least some power to detect expression differences in naïve and/or primed cells. The general conclusions will not be affected, as ESRG will remain dispensable for pluripotency independent of some differently expressed genes. But if ESRG is functional (See point 2 below) it will reveal insights about its potential role.

**Expression analysis by RNA sequencing** | We have more than three clones of ESRG knockout, but we chose and used three lines with unique deletion patterns to demonstrate that each clone is independent. Although we did not use additional knockout lines for the downstream analyses, we confirmed that they could normally expand with PSC-like morphologies. Instead of adding these cell lines for the analysis, as reviewer #2 suggested, we collected RNAs of each line in different passages. We analyzed the global gene expression of (biological triplicates (three lines) x technical triplicates (three passages) = nine samples for each genotype (WT and KO) and each condition (primed and naïve) = 36 samples in total) by RNA sequencing according to the reviewer’s suggestion. As a result, we obtained 36 and 29 of the differentially expressed genes (fold change>2; p-value<0.05) between WT and KO in primed and naïve conditions, respectively. The data have been added in Figure S3 of the revised manuscript. The results do not affect our conclusion but were valuable to confirm our gene expression analysis accuracy.

2. The second important point is to analyse the conservation of ESRG. To judge how expected or unexpected the finding of no phenotype is, it is crucial to know how much evidence is there that ESRG is indeed functional. I.e. is its promotor/gene body more conserved than expected and more or less conserved than other HERV-H elements? Maybe one could even say something about the conservation of its expression in other species from published datasets. In any case, without the most important evidence for the functionality of a genetic element this paper remains very incomplete.

**The conservation analysis of ESRG** | To answer this, we inferred strong selection (divergence) and weak negative selection (human polymorphisms) acting on the exonic sequences of ESRG and contrasted these to its intronic sequences as well as other lincRNAs and protein-coding genes. ESRG has fewer than 1% conserved sites (phastCons>0.9) and no significant differences in the fixation rate in exons compared to introns, thus not showing any signs for a deviation from neutrality. Furthermore, the excess of rare alleles within human populations is considerably lower than that of functional protein-coding sites and similar to synonymous sites and other - conserved and non-conserved - lincRNAs. Hence, there is no compelling evidence for selection based on the sequence analysis. Moreover, the expression analysis of chimpanzee and human iPSCs and a control cell type showed that ESRG is one of the genes with the most diverged expression patterns. We have added the data in Figure 1 of the revised manuscript.

More minor concerns

1. The language is not appropriate as there are way too many errors and/or imprecise usage of language that considerably weakens the impression of precise experiments. Examples include:
   1. Line 102 ff: “that flanked ~8,400 bp of the genomic region including the entire ESRG gene based on the human genome database and RNA-seq data”
   2. Line 107: “in 3 WT versus 3 KO manner”
   3. Line 44: “contribute to reprogram of differentiated cells to pluripotent state”

We have corrected them in the revised manuscript.

2. At the beginning of the results it is not clear which data was generated for this study and how this was done and which data was already published.

We have added the description of accession numbers to use published data in the figure legend.
